# Synthesis, reactivity and biological activity of 5-alkoxymethyluracil analogues

**DOI:** 10.3762/bjoc.7.80

**Published:** 2011-05-26

**Authors:** Lucie Brulikova, Jan Hlavac

**Affiliations:** 1 Department of Organic Chemistry, Faculty of Science, Institute of Molecular and Translational Medicine, Palacky University, 17. Listopadu 12, 771 46 Olomouc, Czech Republic. Tel.: +420 585 634 405; Fax: +420 585 634 465

**Keywords:** 5-alkoxymethyluracil, biological activity, nucleosides

## Abstract

This review article summarizes the results of a long-term investigation of 5-alkoxymethyluracil analogues and is aimed, in particular, at methods of syntheses. Most of the presented compounds were synthesized in order to evaluate their biological activity, therefore, a brief survey of biological activity, especially antiviral, cytotoxic and antibacterial, is also reported.

## Review

### Introduction

Modifications of nucleic acid components play a significant role in the field of nucleic acids research. In particular, nucleoside analogues find broad therapeutic applications in anticancer treatments and antiviral chemotherapy. In anticancer chemotherapy the huge amount of knowledge concerning processes taking place through the cell cycle has enabled researchers to break through and to understand the mechanisms of action of many anticancer agents. 5-Fluorouracil, for instance, was one of the first [1] and most investigated anticancer drugs, either chemically or biologically, and triggered the research of 5-substituted pyrimidine analogues.

The elucidation of the life cycle of a virus is crucial in antiviral chemotherapy. Several 5-substituted pyrimidine analogues capable of affecting the life cycle of viruses were discovered as highly active antiviral agents. Two such drugs with antiviral properties are 5-iodo-2'-deoxyuridine, discovered in the 1960s as the first agent that is active against *Herpes simplex* and *Varicella zoster* viruses [2–3], and 5-vinyl-2'-deoxyuridine, exhibiting high activity against HSV [4–5], which in turn led to studies on the synthesis and biological activity of its analogues.

From these pieces of knowledge, we draw inspiration for the development of new potent biologically active compounds; Compounds that might be more selective, more specific and much less toxic for organisms.

**Figure 1:**
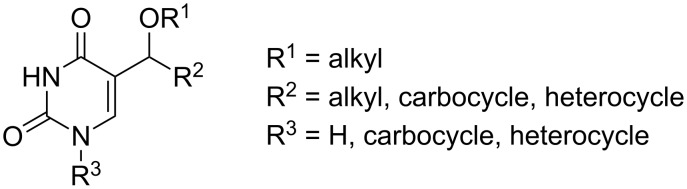
Investigated derivatives.

One of those groups of investigated derivatives is a group of uracil analogues modified at the 5 position by an ether or ester moiety. Since a vast number of C-5 modified pyrimidine analogues are known, this review is focused on a group of selected compounds with specific substituents (Figure 1) and most attention is paid to the studies on synthesis of selected derivatives. A brief survey of the biological activity of investigated compounds is also reported. The following chapters concerning the synthesis are arranged according to the products of synthetic routes.

### Synthesis

#### Synthesis of alkoxy-haloalkyl derivatives

**Figure 2:**
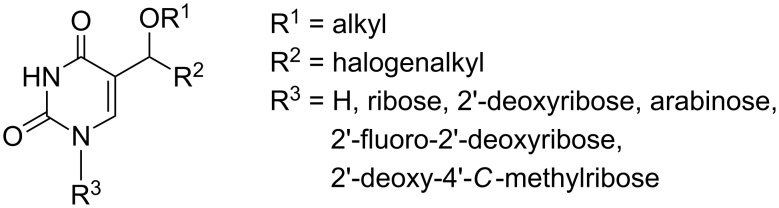
Modifications of uracil ring.

The most numerous and also the most investigated group of the above mentioned derivatives is a group of alkoxy-haloalkyl compounds derived either from uracil or nucleosides (Figure 2). With regard to the high variability of sugar moiety, the description of all the compounds is divided into sections according to the nature of the furanose present.

**Figure 3:**
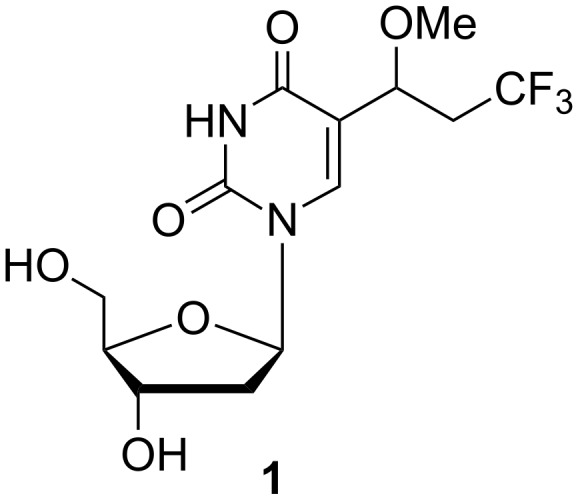
5-(3,3,3-Trifluoro-1-methoxypropyl)-2'-deoxyuridine (**1**).

**2'-Deoxyuridine analogues:** The earliest article describing 2'-deoxyuridine analogues was focused on uracil analogues modified at position 5 by a fluorine containing moiety [6]. Bases or nucleosides substituted by fluorine have been investigated as potent anticancer agents since the 1960s. Nevertheless, many such modified compounds were also synthesized in order to investigate their antiviral activity. As a consequence of interest in biologically active fluoro derivatives, Bergstrom and co-workers carried out the synthesis of 5-(3,3,3-trifluoro-1-methoxypropyl)-2'-deoxyuridine (**1**) (Figure 3) which was the first perfluoro derivative from the group of aforementioned compounds.

**Scheme 1:**
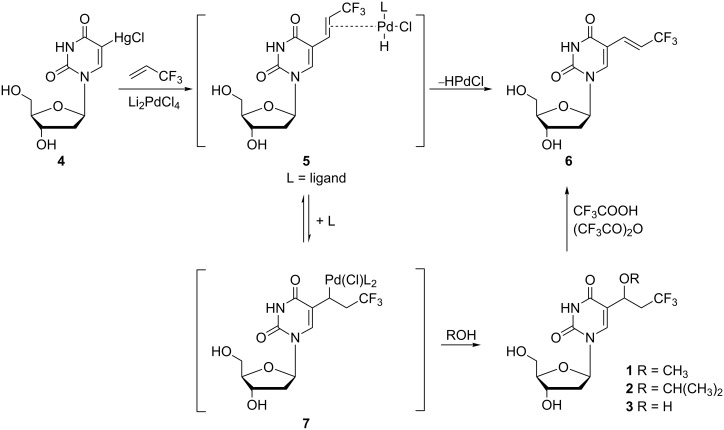
Synthesis of 5-(3,3,3-trifluoro-1-methoxypropyl)-2'-deoxyuridine (**1**) and 5-(3,3,3-trifluoro-1-(2-propyloxy)prop-1-yl)-2'-deoxyuridine (**2**).

The synthetic route to the desired fluoro compound **1** utilized the known reaction [7] between the organomercuri intermediate, 5-chloromercuri-2'-deoxyuridine, and a palladium catalyst. The reaction, carried out in methanol, afforded 17% of *(E)*-5-(3,3,3-trifluoro-1-propenyl)-2'-deoxyuridine (**6**) and 36% of 5-(3,3,3-trifluoro-1-methoxypropyl)-2'-deoxyuridine (**1**) (Scheme 1).

Shortly after publishing the successful synthesis of trifluoro nucleoside **1**, Bergstrom and co-workers reported a presumed mechanism for its formation (Scheme 1) [8]. In addition, they also focused their research on isopropyloxy analogue **2**.

Reaction of 3,3,3-trifluoropropene with 5-chloromercuri-2'-deoxyuridine (**4**) in methanol gave two major products *(E)*-5-(3,3,3-trifluoro-1-propenyl)-2'-deoxyuridine (**6**) and derivative **1** in an approximately 1:2 ratio. The authors also carried out the synthesis in other solvents, such as *N,N*-dimethylformamide, 2-propanol or acetonitrile, and found that the use of solvents other than methanol led to decreased yields of the C-5 substituted products. The utilization of 2-propanol, for instance, afforded unsaturated derivative **6** in 8% yield and 5-(3,3,3-trifluoro-1-(2-propyloxy)prop-1-yl)-2'-deoxyuridine (**2**) in 12% yield. Moreover, fluoro compound **1** can be converted to the propenyl derivative **6** by treating with a mixture of trifluoroacetic acid and trifluoroacetic acid anhydride. Interestingly, a third product was isolated from the reaction mixture when similar reactions of 5-chloromercuri-2'-deoxyuridine (**4**) with 3,3,3-trifluoropropene were carried out. This was shown to be 5-(3,3,3-trifluoro-1-hydroxypropyl)-2'-deoxyuridine (**3**) which was obtained in 38–40% yield, however, the authors were unable to account for its formation.

**Scheme 2:**
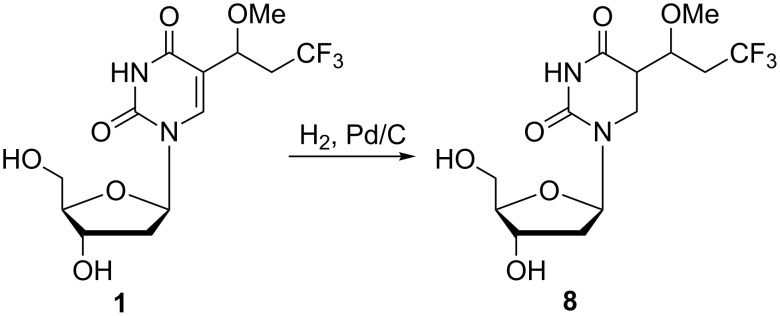
Synthesis of 5-(3,3,3-trifluoro-1-methoxyprop-1-yl)-5,6-dihydro-2'-deoxyuridine (**8**).

An attempt at hydrogenolysis of the methoxy group of derivative **1** using H_2_ and Pd/C afforded 5-(3,3,3-trifluoro-1-methoxyprop-1-yl)-5,6-dihydro-2'-deoxyuridine (**8**) indicating that ring reduction occurred instead of hydrogenolysis (Scheme 2).

**Scheme 3:**
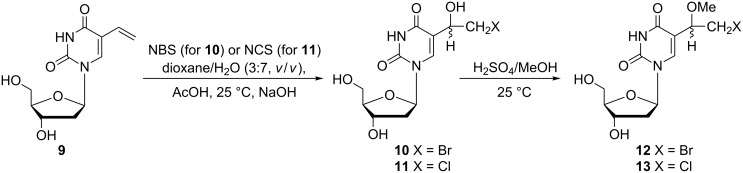
Synthesis of 5-(methoxy-2-haloethyl)-2'-deoxyuridines **12** and **13**.

The synthesis of a large range of alkoxyhalogenalkyl 2'-deoxyuridine nucleosides was successfully performed by Kumar and co-workers over the years 1989–1994 [9–13]. Almost all of these compounds were synthesized in order to evaluate their biological activity and especially their antiviral activity. In 1989 Kumar and co-workers reported, amongst other things, two 5-(1-methoxy-2-haloethyl)-2'-deoxyuridines **12** and **13** (Scheme 3) [9].

Their synthesis was based on the addition of HOX (X = Br, Cl) to the vinyl moiety of 5-vinyl-2'-deoxyuridine (**9**). The reaction was carried out in aqueous dioxane, and hydroxybromoethyl **10** and hydroxychloroethyl **11** derivatives were obtained in 70% and 60% yields, respectively. Subsequent treatment of hydroxyl derivatives **10** and **11** with methanolic sulfuric acid gave the corresponding desired 5-(1-methoxy-2-haloethyl) derivatives **12** and **13** in 93 and 98% yields, respectively. No details of the separation method for the two diastereomers were described in their article.

**Scheme 4:**
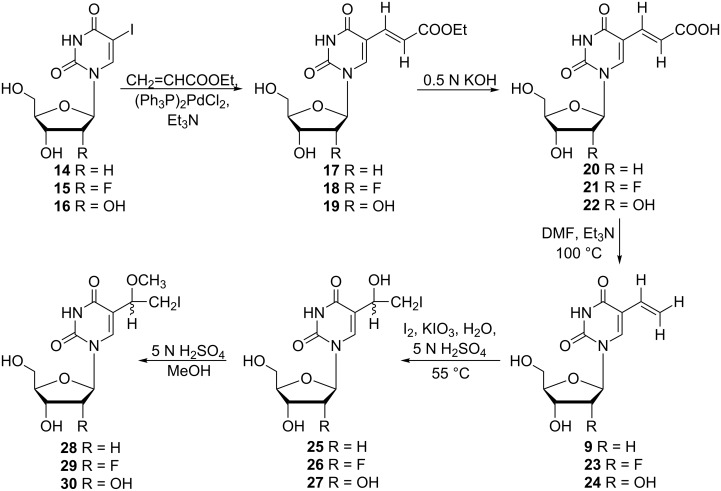
Synthesis of 5-(1-methoxy-2-iodoethyl) nucleosides **28**–**30**.

A year later, Kumar and co-workers extended their research to the modification of the sugar portion of nucleosides [10] by preparing the iodomethoxy derivatives of 2'-deoxyuridine **28**, 2'-fluoro-2'-deoxyuridine **29** and uridine **30** (Scheme 4).

The authors utilized the known palladium acetate-triphenylphosphine-catalyzed reaction of 5-iodo-2'-deoxyuridine with vinyl acetate for the preparation of 5-vinyl-2'-deoxyuridine (**9**) [14]. However, attempts to prepare 2'-fluoro-2'-deoxyuridine **23** and uridine analogue **24** by this method were unsuccessful. Hence, the 5-vinyl derivatives **9**, **23** and **24** were prepared by three-step palladium-catalyzed synthesis of 5-iodo-2'-fluoro-2'-deoxyuridine (**15**) and 5-iodouridine (**16**) with ethyl acrylate, followed by subsequent alkaline hydrolysis and decarboxylation. Iodination of 5-vinyl analogues **9**, **23** and **24** with iodine in the presence of the iodic acid as an oxidizing agent afforded 5-(1-hydroxy-2-iodoethyl)-2'-deoxyuridine (**25**, 59%), 5-(1-hydroxy-2-iodoethyl)-2'-fluoro-2'-deoxyuridine (**26**, 72%) and 5-(1-hydroxy-2-iodoethyl)uridine (**27**, 65%) as diasteroisomeric mixtures. Finally, treatment of hydroxy derivatives **25**–**27** with methanolic sulfuric acid gave the desired 5-(1-methoxy-2-iodoethyl) nucleosides **28**–**30** in 81–94% yields. All three compounds were obtained as mixtures of two diastereomers.

**Figure 4:**
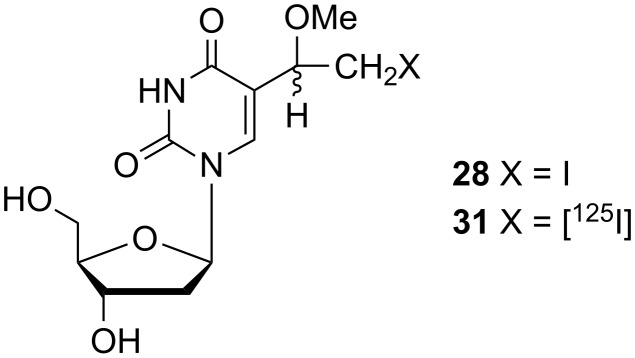
[^125^I] radiolabelled 5-(1-methoxy-2-iodoethyl)-2'-deoxyuridine **31**.

In order to develop new potential tumor localization agents, Iwashina and co-workers investigated the radiolabelled 5-(1-methoxy-2-iodoethyl) nucleoside **31** (Figure 4) [15] which was obtained by radio-iodination of 5-(1-methoxy-2-iodoethyl)-2'-deoxyuridine (**28**) via isotope exchange by the pivalic acid melt method.

**Scheme 5:**
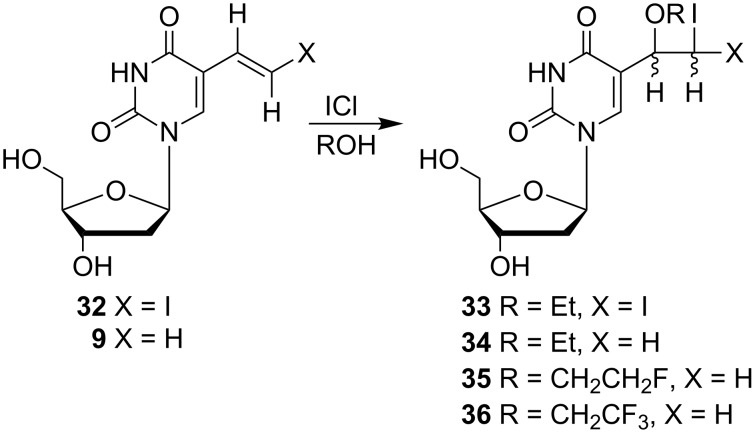
Synthesis of 5-(1-alkoxy-2-iodoethyl) **34**–**36** and 5-(1-ethoxy-2,2-diiodoethyl)-2'-deoxyuridine (**33**).

In addition to the above mentioned 5-(1-methoxy-2-iodoethyl) nucleosides, Kumar and co-workers also reported the synthesis of other alkoxy derivatives – 5-(1-alkoxy-2-iodoethyl) and 5-(1-ethoxy-2,2-diiodoethyl)-2'-deoxyuridine analogues **33**–**36** (Scheme 5) [11]. The reaction of (*E*)-5-(2-iodovinyl) **32** and 5-vinyl-2'-deoxyuridine (**9**) with iodine monochloride and alcohols such as ethanol, 2-fluoroethanol or 2,2,2-trifluoroethanol afforded 5-(1-ethoxy-2,2-diiodoethyl) **33** and 5-(1-alkoxy-2-iodoethyl)-2'-deoxyuridines **34**–**36**, in 33–90% yields. All of these four products **33**–**36** were obtained as a mixture of two diastereomers in a 1:1 ratio.

**Scheme 6:**
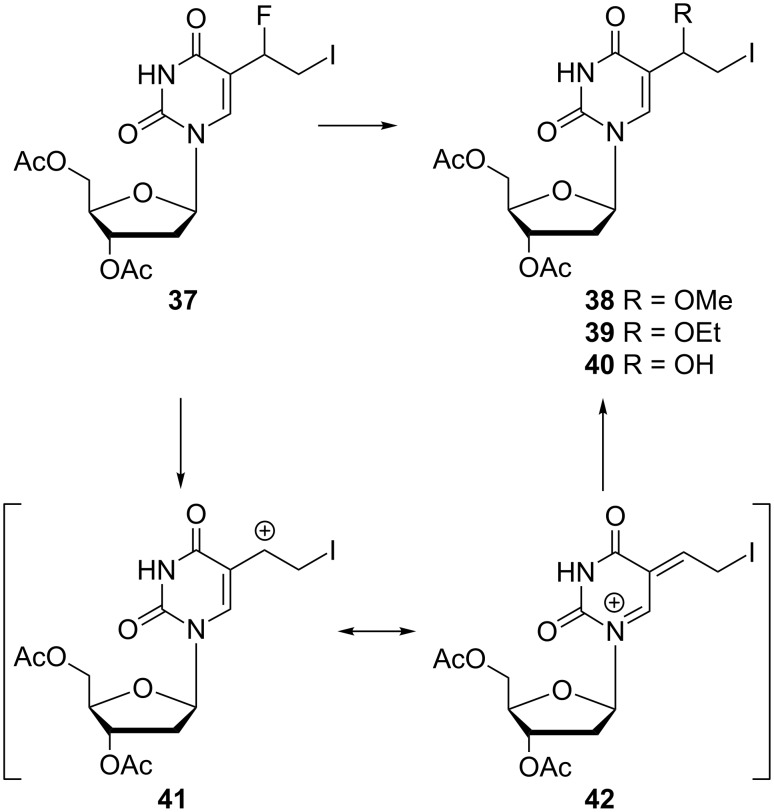
Synthesis of 5-(1-methoxy-2-iodoethyl)-3',5'-di-*O*-acetyl-2'-deoxyuridine (**38**) and 5-(1-ethoxy-2-iodoethyl)-3',5'-di-*O*-acetyl-2'-deoxyuridine (**39**).

In the synthesis of 5-(1-fluoro-2-iodoethyl)-3',5'-di-*O*-acetyl-2'-deoxyuridine (**37**), by-products such as 5-(1-methoxy-2-iodoethyl)-3',5'-di-*O*-acetyl-2'-deoxyuridine (**38**) and 5-(1-ethoxy-2-iodoethyl)-3',5'-di-*O*-acetyl-2'-deoxyuridine (**39**) were identified (Scheme 6) [12]. From the reaction of 5-(1-hydroxy-2-iodoethyl)-3',5'-di-*O*-acetyl-2'-deoxyuridine (**40**) with DAST (Et_2_NSF_3_) at −40 °C in anhydrous dichloromethane, 5-(1-fluoro-2-iodoethyl)-3',5'-di-*O*-acetyl-2'-deoxyuridine (**37**) as well as 5-(1-methoxy-2-iodoethyl)-3',5'-di-*O*-acetyl-2'-deoxyuridine (**38**) and 5-(1-ethoxy-2-iodoethyl)-3',5'-di-*O*-acetyl-2'-deoxyuridine (**39**) were obtained as major products. The authors suggested a mechanism for the formation of methoxy **38** and ethoxy **39** derivatives, which was based on decomposition of 5-(1-fluoro-2-iodoethyl)-3',5'-di-*O*-acetyl-2'-deoxyuridine (**37**) to carbonium cation intermediates **41** and **42** at 25 °C. Subsequent reaction of cation **41** with methanol, ethanol or water produced the alkoxy derivatives **38** and **39**. The authors presumed that the nucleosides **38** and **39** were formed during the silica gel column chromatography, where a mixture of methanol, chloroform and ethanol was used as eluent.

**Figure 5:**
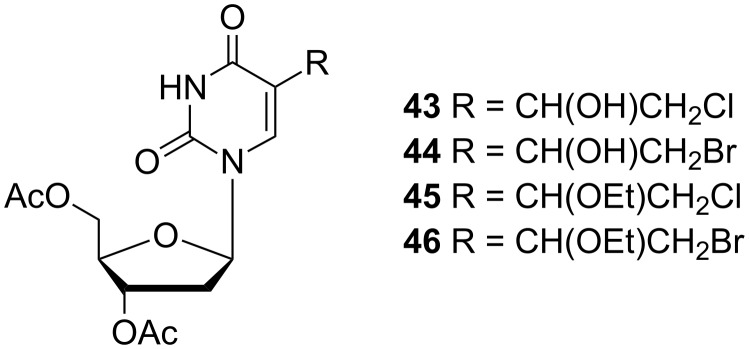
5-(1-Hydroxy(or ethoxy)-2-haloethyl)-3',5'-di-*O*-acetyl-2'-deoxyuridines **43**–**46**.

In addition, the authors described the reaction of the 5-(1-hydroxy-2-chloroethyl) **43** and 5-(1-hydroxy-2-bromoethyl)-3',5'-di-*O*-acetyl-2'-deoxyuridine (**44**) with thionyl bromide, which provided 5-(1-ethoxy-2-chloroethyl)-3',5'-di-*O*-acetyl-2'-deoxyuridine (**45**) and 5-(1-ethoxy-2-bromoethyl)-3',5'-di-*O*-acetyl-2'-deoxyuridine (**46**) (Figure 5).

**Scheme 7:**
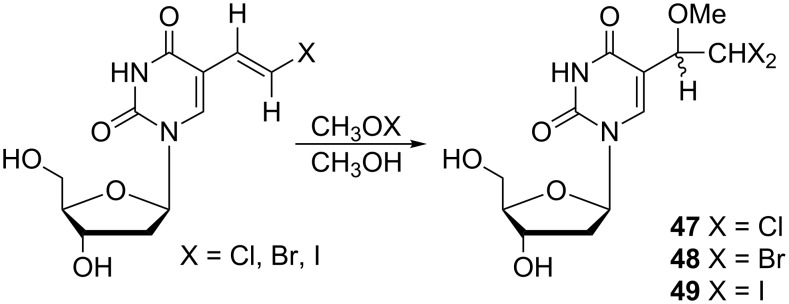
5-(1-Methoxy-2,2-dihaloethyl)-2'-deoxyuridines **47**–**49**.

The introduction of an additional halogen to the ethyl moiety at the C-5 position of the uracil base led to dihalo derivatives that were also reported by Kumar and co-workers [13]. The required 5-(1-methoxy-2,2-dihaloethyl)-2'-deoxyuridines **47**–**49** (Scheme 7) were prepared by the addition of CH_3_OX (X = Cl, Br or I) to the vinyl moiety of (*E*)-5-(2-halovinyl)-2'-deoxyuridine.

**Scheme 8:**
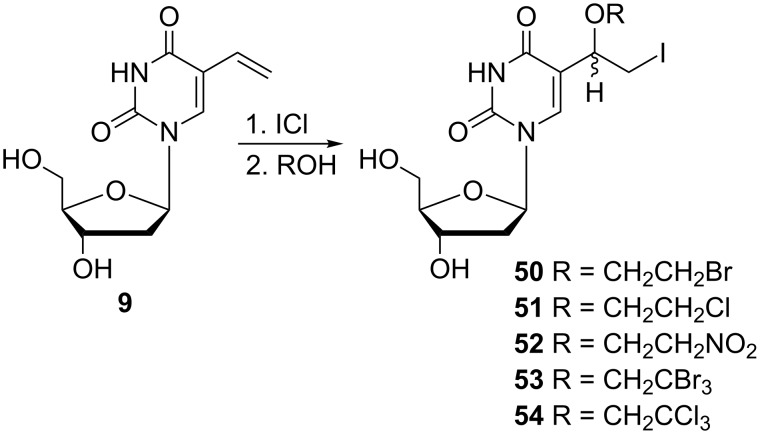
Synthesis of 5-[1-(2-haloethyl(or nitro)ethoxy)-2-iodoethyl]-2'-deoxyuridines **50**–**54**.

Rai and co-workers developed an efficient synthesis of 5-[1-(2-halo(or nitro)ethoxy)-2-iodoethyl]-2'-deoxyuridines **50**–**54** (Scheme 8) and evaluated their antiviral activity [16]. For this purpose, 5-vinyl-2'-deoxyuridine (**9**) was used as the starting compound. The regiospecific reaction of the 5-vinyl-2'-deoxyuridine (**9**) with iodine monochloride in the presence of various alcohols afforded 2'-deoxynucleosides **50**–**54** in 24–52% yield.

**Scheme 9:**
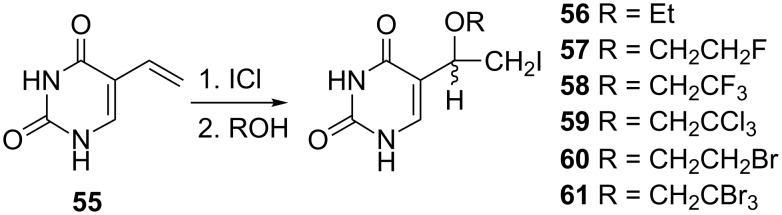
Synthesis of alkoxyuracil analogues **56**–**61**.

**Uracil analogues:** The syntheses of some of the aforementioned 2'-deoxyuridine analogues were also described for modified uracil derivatives. The first group of these derivatives is represented by alkoxyiodoethyl derivatives **56**–**59** prepared by the reaction of 5-vinyluracil (**55**) with iodine monochloride (Scheme 9) [11]. The reaction was carried out in the presence of ethanol, 2-fluoroethanol, 2,2,2-trifluoroethanol or 2,2,2-trichloroethanol to give the 5-(1-ethoxy-2-iodoethyl) **56**, 5-[1-(2-fluoroethoxy)-2-iodoethyl] **57**, 5-[1-(2,2,2-trifluoroethoxy)-2-iodoethyl] **58** and 5-[1-(2,2,2-trichloroethoxy)-2-iodoethyl] **59** uracil analogues.

Rai and co-workers also used the same procedure to prepare a series of 5-[1-(2-haloethoxy-2-iodoethyl)]uracils **60**–**61** (Scheme 9) [16]. Thus, regiospecific addition of iodine monochloride in the presence of various alcohols to 5-vinyluracil (**55**) gave 5-[1-(2-bromoethoxy)-2-iodoethyl]uracil (**60**) and 5-[1-(2,2,2-tribromoethoxy)-2-iodoethyl)]uracil (**61**).

**Figure 6:**
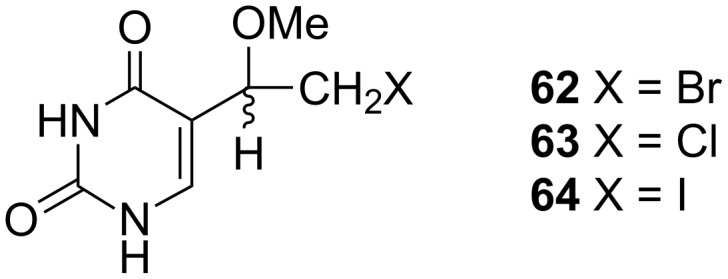
5-(Methoxy-2-haloethyl)uracils **62**–**64**.

A similar reaction was used 14 years earlier by Kumar and co-workers for the synthesis of 5-(1-methoxy-2-haloethyl)uracils **62**–**64** (Figure 6) [17] by the addition of HOX (X = Br, Cl) or ICl to the 5-vinyluracil (**55**) and subsequent treatment with methanolic sulfuric acid.

**Scheme 10:**
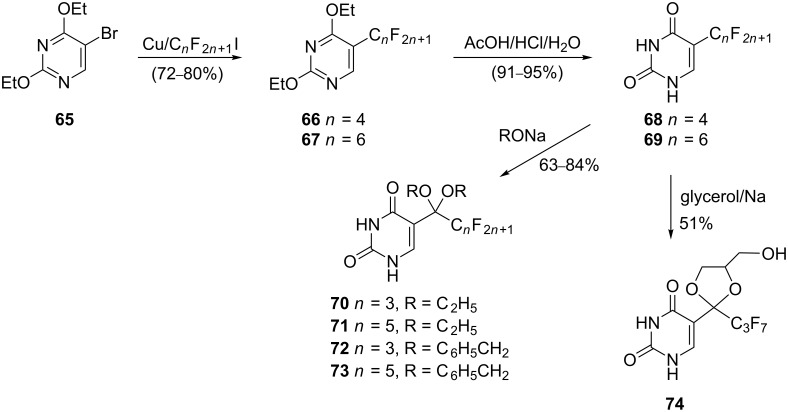
Synthesis of perfluoro derivatives **70**–**74**.

In 2002 Ismail and co-workers published an efficient synthetic route for the preparation of ethoxy-substituted 5-(perfluoroalkyl)pyrimidines (Scheme 10) and investigated their regioselective transformations [18]. Some of these fluorine-containing pyrimidine analogues are potent antitumor and antiviral agents.

The reported synthesis started with the treatment of 5-bromo-2,4-diethoxypyrimidine (**65**) with either perfluorobutyl or perfluorohexyl iodide in the presence of activated copper bronze in DMSO. This reaction afforded 5-(perfluoroalkyl)pyrimidines **66** and **67** in high yields. Subsequent acid hydrolysis of **66** and **67** provided 5-(perfluoroalkyl)pyrimidines **68** and **69**. The latter readily underwent nucleophilic attack by alkoxide ions to yield alicyclic or cyclic acetals **70**–**73** and **74**, respectively, depending on the alcohol used.

**Scheme 11:**
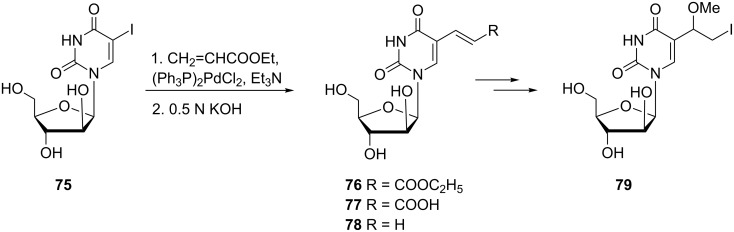
Synthesis of 1-β-D-arabinofuranosyl-5-(1-methoxy-2-iodoethyl)uracil (**79**).

**Uridine and arabinofuranosyl analogues:** 5-Substituted uracil nucleosides where the sugar component is ribose or arabinose have also been prepared. Johar and co-workers described the synthesis of 1-β-D-arabinofuranosyl-5-(1-methoxy-2-iodoethyl)uracil (**79**) (Scheme 11) in a recent article [19]. The compound was also reported by Kumar and co-workers in 1992 [20].

The synthesis was based on the reaction of 1-β-D-arabinofuranosyl-5-iodouracil (**75**) with ethyl acrylate in the presence of Pd catalyst and TEA. Subsequent alkaline hydrolysis of **76** afforded the (*E*)-5-(2-carboxyvinyl) derivative **77**. Decarboxylation of the latter gave 5-vinyl-arabinouridine **78** which was reacted with iodine in the presence of an oxidizing agent, iodic acid to afford 1-β-D-arabinofuranosyl-5-(1-hydroxy-2-iodoethyl)uracil. Treatment of the hydroxyl derivative with methanolic sulfuric acid gave the required methoxy nucleoside **79**.

**Scheme 12:**
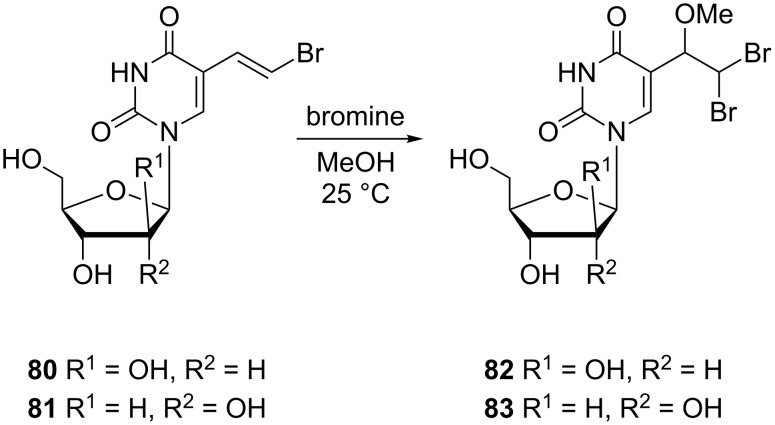
Synthesis of 1-β-D-arabinofuranosyl-5-(2,2-dibromo-1-methoxyethyl)uracil **82** and uridine analogue **83**.

The regiospecific addition of bromine in methanol to (*E*)-5-(2-bromovinyl)arabinouridine **80** and its uridine counterpart **81** afforded 1-β-D-arabinofuranosyl-5-(2,2-dibromo-1-methoxyethyl)uracil (**82**) and the *ribo* analogue **83** (Scheme 12) [21].

**Scheme 13:**
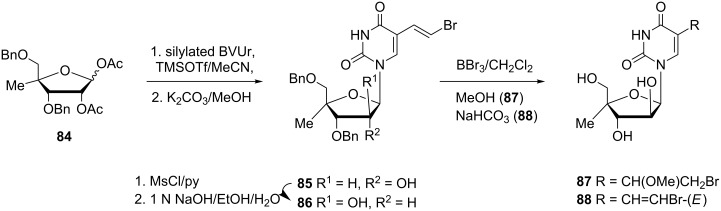
Synthesis of methoxy derivative **87**.

In a search for new antiviral agents, 4'-*C*-methylpyrimidine nucleosides were synthesized (Scheme 13) and their biological activity evaluated [22]. Firstly, the 4'-*C*-methyl-D-ribose **84** was prepared by a previously described procedure [23]. Next 5-bromovinyluracil (BVUr) was silylated and reacted with **84** in the presence of TMSOTf as the Lewis acid. This was followed by deacetylation with anhydrous K_2_CO_3_ in MeOH to provide the di-*O*-benzylated nucleoside **85** in 73% yield. For the change of the configuration at 2'-C, derivative **85** was converted to its mesylate and treated with NaOH in EtOH-H_2_O to afford 4'-*C*-methylnucleoside **86** in 58% yield. Finally, nucleoside **86** was debenzylated with BBr_3_ in CH_2_Cl_2_ at −78 °C. On quenching of the reaction with MeOH the unexpected formation of methoxy derivative **87** was observed, whilst quenching with saturated NaHCO_3_ solution gave the target derivative **88**.

#### Synthesis of alkoxy-azidoalkyl derivatives

**Scheme 14:**
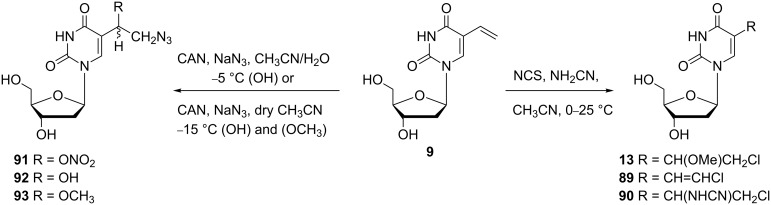
Synthesis of 5-(1-methoxy-2-azidoethyl)-2'-deoxyuridine (**93**).

In addition to the reactions of HOX or CH_3_OX (X = Cl, Br, I) with 5-vinyl-2'-deoxyuridine (**9**), Kumar and co-workers reported the regiospecific addition of halogenocyanamides (X-NHCN) to **9** to produce 5-(1-cyanamido-2-chloroethyl)-2'-deoxyuridine (**90**, Scheme 14) [24].

In addition to **90**, the reaction of 5-vinyl-2'-deoxyuridine (**9**) with *N*-chlorosuccinimide (NCS) and cyanamide was accompanied by the formation of a mixture of by-products such as (*E*)-5-(2-chlorovinyl)-2'-deoxyuridine (**89**) and 5-(1-methoxy-2-chloroethyl)-2'-deoxyuridine (**13**). 5-Vinyl-2'-deoxyuridine (**9**) can also undergo reaction with ceric ammonium nitrate (CAN) and sodium azide in aqueous acetonitrile to give 5-(1-hydroxy-2-azidoethyl)-2'-deoxyuridine (**92**) in 32% yield. When dry acetonitrile was used as the reaction solvent and the reaction was quenched with methanol, 5-(1-methoxy-2-azidoethyl)-2'-deoxyuridine (**93**) was obtained in 25% yield.

#### Synthesis of alkoxy-alkyl derivatives

The C-5 modified pyrimidine nucleosides with the short alkyl substituent have been at the center of intense interest since the early 1970s due to their potential chemotherapeutic and antiviral properties. It was reported, for instance, that 5-ethyluracil may undergo incorporation into bacterial DNA [25] and that 5-ethyl-2'-deoxyuridine readily replaces thymidine in bacteriophage DNA [26].

**Scheme 15:**
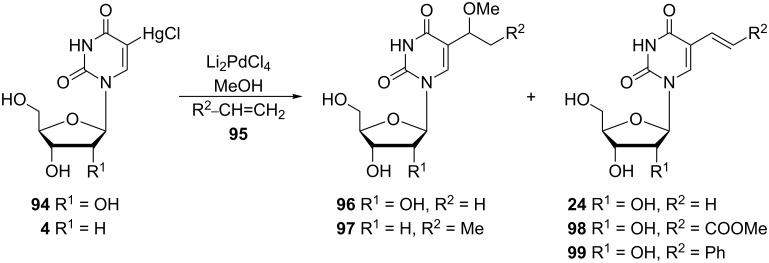
Synthesis of methoxyalkyl derivatives **96** and **97**.

Bergstrom and co-workers also targeted the alkyl modification at position 5 of pyrimidine analogues [27] and synthesized 5-(1-methoxyethyl)uridine (**96**) (Scheme 15) with a view towards transformation of the latter to the 5-ethyl analogue. In their reaction the organomercuri nucleoside **94** was converted to the organopalladium analogue via the reaction with 0.1 M palladium catalyst and ethene in methanol. Surprisingly, the major product of the reaction was the methoxy derivative **96** in 39% yield instead of the expected 5-vinyluridine (**24**). Similarly, the 2'-deoxyuridine organomercuri derivative **4** reacted with propene in the presence of Li_2_PdCl_4_ in methanol to give 5-(1-methoxypropyl)-2'-deoxyuridine (**97**) as one of the products but the compound was not separated from the reaction mixture.

**Scheme 16:**
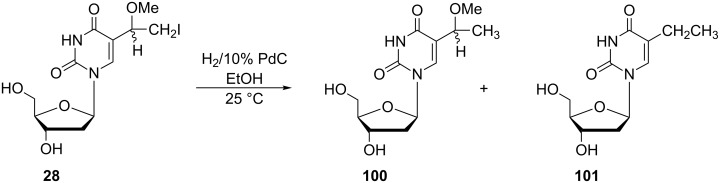
Synthesis of 5-(1-methoxyethyl)-2'-deoxyuridine (**100**).

5-(1-Methoxyethyl)-2'-deoxyuridine (**100**) was prepared by Kumar and co-workers. However, they reported a different synthetic route leading to the desired nucleoside **100**, which differs from the above mentioned preparation of uridine analogue **96** (Scheme 16) [28]. 5-(1-Methoxy-2-iodoethyl)-2'-deoxyuridine (**28**) was reacted with hydrogen in the presence of 10% Pd/C in ethanol at 25 °C to give as the major product 5-(1-methoxyethyl)-2'-deoxyuridine (**100**) in 26% yield accompanied by 5-ethyl-2'-deoxyuridine (**101**) in 13% yield.

**Scheme 17:**
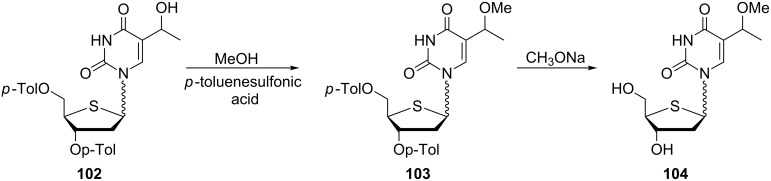
Synthesis of 2'-deoxy-5-(1-methoxyethyl)-4'-thiouridine (**104**).

Because of the high activity of (*E*)-5-(2-bromovinyl)-2'-deoxy-4'-thiouridine against HSV-1, HSV-2 and *Varicella zoster virus*, the group of 2'-deoxy-4'-thionucleosides have been extensively investigated [29]. In this context, a series of 5-substituted 2'-deoxy-4'-thiopyrimidine nucleosides were synthesized by Rahim and co-workers in order to evaluate their antiviral activity [30]. One such compound is 2'-deoxy-5-(1-methoxyethyl)-4'-thiouridine (**104**) (Scheme 17). The desired methyl ether **103** was obtained by the methylation of 2'-deoxy-3',5'-di-*O*-*p*-toluoyl-5-(1-hydroxymethyl)-4'-thiouridine (**102**) with methanol in the presence of *p*-toluenesulfonic acid. Subsequent treatment with sodium methoxide gave deprotected thiouridine **104**.

**Figure 7:**
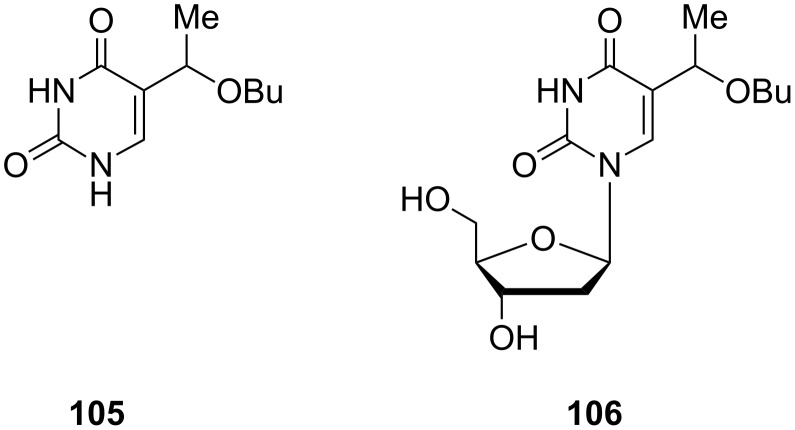
5-(1-Butoxyethyl)uracil **105** and 5-(1-butoxyethyl)-2'-deoxyuridine (**106**).

Among others, Jones and co-workers focused their attention on alkyl ethers with longer chains [31]. From a study of some chemical properties of 5-vinyluracil, they successfully synthesized 5-(1-butoxyethyl)uracil (**105**) and 5-(1-butoxyethyl)-2'-deoxyuridine (**106**) (Figure 7). When 2'-deoxy-5-vinyluridine was reacted with butan-1-ol in the presence of trifluoroacetic acid at 55 °C, a mixture of diastereomers of 5-(1-butoxyethyl)-2'-deoxyuridine (**106**) was obtained. When nearly saturated HCl in dioxane at 75 °C was used, only traces of nucleoside **106** were formed and 5-(1-butoxyethyl)uracil (**105**) was obtained as a major product.

**Scheme 18:**
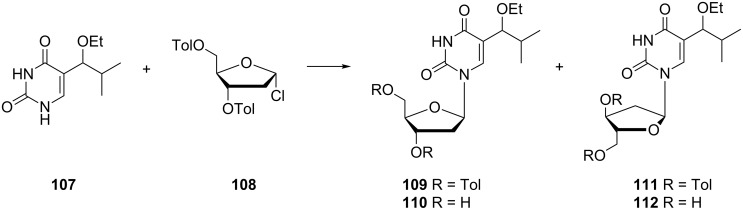
Synthesis of β- and α-anomer of 5-(1-ethoxy-2-methylprop-1-yl)-2'-deoxyuridine.

Other changes at the C-5 position of pyrimidine analogues led to the anomeric 5-alkyl derivatives **110** and **112,** which were synthesized in the early 1980s (Scheme 18) [32]. This preparation was based on the condensation of 5-(1-ethoxy-2-methylprop-1-yl)uracil (**107**) with 2-deoxy-3,5-di-*O*-toluoyl-α-D-ribofuranosyl chloride (**108**). Initially, the uracil ring was protected by the silylation with hexamethyldisilazane. Subsequently, this modified uracil was reacted with protected 2-deoxyribose in the presence of SnCl_4_. Finally, protected α- and β-anomers **111** and **109** were treated with methanolic sodium methoxide to afford the nucleosides **112** and **110**.

#### Synthesis of acyloxy derivatives

**Scheme 19:**
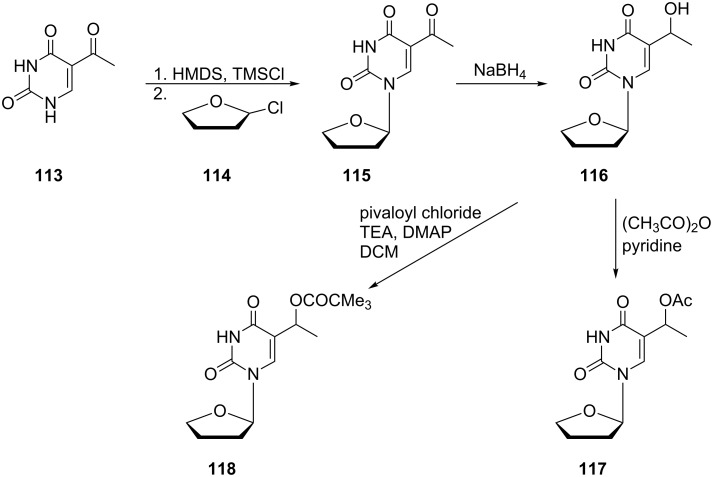
Synthesis of 5-(1-acyloxyethyl)-1-(tetrahydrofuran-2-yl)uracils **117** and **118**.

The substitution at position 5 of the pyrimidine ring by acyloxy moiety provides another group of derivatives. Some of these compounds were synthesized as 1-(tetrahydrofuran-2-yl) pyrimidine analogues [33]. The use of such an atypical furanose ring avoids complications with the protection of hydroxyl groups of 2'-deoxyribose during the development of an appropriate method for acylation of the side chain hydroxyl group. The acyloxy derivatives **117** and **118** were synthesized in only a few steps (Scheme 19).

Firstly, 5-acetyluracil (**113**) was silylated with hexamethyldisilazane containing trimethylsilyl chloride and the silylated acetyluracil coupled with 2-chlorotetrahydrofuran (**114**) to afford 5-acetyl-1-(tetrahydrofuran-2-yl)uracil (**115**). Subsequent reduction of the keto group with sodium borohydride gave 5-(1-hydroxyethyl)-1-(tetrahydrofuran-2-yl)uracil (**116**). Final acetylation of the hydroxyl group of derivative **116** with acetic anhydride in pyridine afforded 5-(1-acetyloxyethyl)-1-(tetrahydrofuran-2-yl)uracil (**117**), whilst treatment of **116** with pivaloyl chloride in the presence of triethylamine and *N,N*-dimethylaminopyridine gave the pivalate ester **118**.

**Scheme 20:**
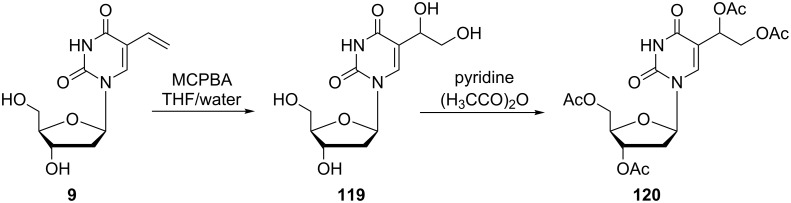
Synthesis of 5-(1,2-diacetoxyethyl)-3',5'-di-*O*-acetyl-2'-deoxyuridine **120**.

An oxidation of 5-vinyl-2'-deoxyuridine (**9**) was also studied (Scheme 20) [31]. The authors used *m*-chloroperbenzoic acid as an oxidizing agent and observed its influence on the reactivity of the vinyl substituent in the presence and absence of water. When the reaction is performed in the absence of water an epoxide should be obtained. Nevertheless, the authors instead observed a ring opening. However, the product was not fully characterized. As long as water was used, 2'-deoxy-5-(1,2-dihydroxyethyl)uridine (**119**) was obtained. This dihydroxy derivative **119** was characterized after the transformation to the acetyl analogue **120** using acetic anhydride in pyridine.

#### Synthesis of arylderivatives

**Scheme 21:**
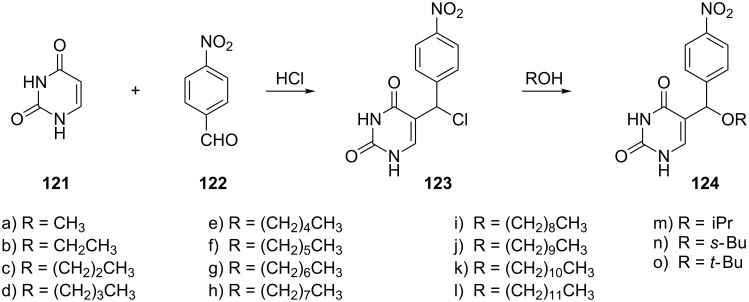
Synthesis of 5-[alkoxy-(4-nitrophenyl)methyl]uracils **124**.

The synthesis of 5-[alkoxy-(4-nitrophenyl)methyl]uracils **124** (Scheme 21) has recently been investigated [34]. The authors reported the synthesis of alkoxy derivatives with alkyl chain lengths C_1_-C_12_ whose preparation started with the condensation reaction of uracil and *p*-nitrobenzaldehyde in concentrated hydrochloric acid. Subsequently, the resulting 5-[chloro-(4-nitrophenyl)methyl]uracil (**123**) was reacted with different alcohols to give the corresponding ethers **124a**–**o**.

**Scheme 22:**
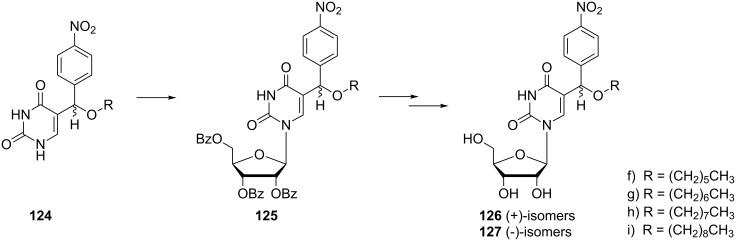
Synthesis of 5-[alkoxy-(4-nitrophenyl)methyl]uridines **126** and **127**.

The introduction of a sugar moiety to the selected analogues **124f**–**i** afforded 5-[alkoxy(4-nitrophenyl)methyl]uridines **126f**–**i** and **127f**–**i** (Scheme 22) [35]. In a first step, the alkoxy uracils **124** were silylated and then reacted with a protected ribose in the presence of TMSOTf to afford diastereomeric mixtures of nucleosides **125**. Diasteroisomers were separated and finally treated with methanolic ammonia to afford nucleosides **126** and **127**.

#### Synthesis of oligonucleotide intermediates

**Scheme 23:**
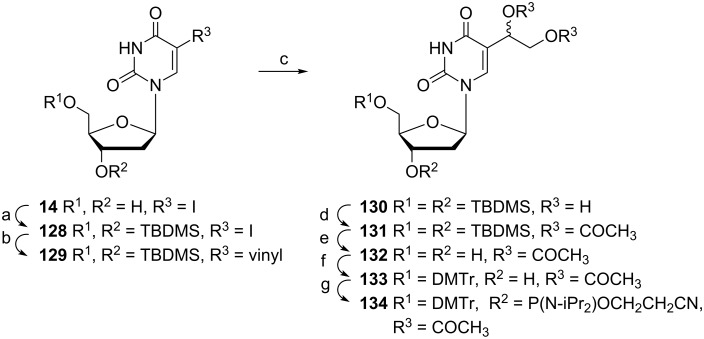
Synthesis of phosphoramidite **134**. **Reaction conditions 1:** (a) TBDMSCl, imidazole, pyridine, 33 h, 99%; (b) vinyl acetate, Pd(OAc)_2_, PPh_3_, Et_3_N, DMF, 70 °C, 16 h, 68%; (c) OsO_4_, 4-methylmorpholine-*N*-oxide, acetone H_2_O *t*-BuOH (4:1:1), 15 h, 44%; (d) Ac_2_O, pyridine, 44 h, 96%; (e) TBAF, THF, 14 h, 75%; (f) DMTrCl, DMAP, Et_3_N, pyridine, 22 h, 78%; (g) [(iPr)_2_N]_2_POCH_2_CH_2_CN, tetrazole, 2.5 h, quant. **Reaction conditions 2:** (a) TBDMSCl, imidazole, DMF, over night; (b) 5 mol % Pd(MeCN)_2_Cl_2_, Bu_3_SnCH=CH_2_ (1.5 equiv), MeCN, 80 °C; (c) cat. OsO_4_, NMO (2.5 equiv), acetone H_2_O *t*-BuOH (4:1:1); (d) Ac_2_O (4 equiv), pyridine; (e) TBAF (3 equiv), AcOH (2 equiv), THF; (f) DMTrCl (1.5 equiv), pyridine; (g) [(iPr)_2_N]_2_POCH_2_CH_2_CN (1.8 equiv), DCI (0.7 equiv), MeCN CH_2_Cl_2_ (1:10).

Modified oligonucleotides are powerful tools in nucleic acid research and their synthesis has become an important aspect of bioorganic and medicinal chemistry. One part of oligonucleotide chemistry associated with this review is focused on the studies of the action of 5-formyl-2'-deoxyuridine, which is one of the oxidative thymidine lesions of DNA formed by ionizing radiation. Consequently, several methods for the preparation of appropriate intermediates for the synthesis of oligodeoxynucleotides containing 5-formyl-2'-deoxyuridine have been published. Sugiyama and co-workers reported a seven-step synthesis of phosphoramidite **134** (Scheme 23, reaction conditions 1) starting with readily available 5-iodo-2'-deoxyuridine (**14**) [36]. The first two steps of the synthesis involved the protection of 3',5'-dihydroxyl groups with the TBDMS group followed by a Pd-catalyzed coupling reaction with vinyl acetate to give the protected 5-vinyluridine **129** in 68% yield. Oxidation with OsO_4_ with subsequent acetylation with acetic anhydride in pyridine gave nucleoside **131**. The target phosphoramidite **134** was obtained after the standard phosphoramidite synthesis starting with the protection of the 5'-OH group with dimethoxytrityl chloride and final phosphitylation.

Later, Kittaka and co-workers reported the synthesis of phosphoramidite **134** under different conditions [37]. The protected 5-iodo-2'-deoxyuridine **128** was subjected to a Stille coupling reaction with tributyl(vinyl)tin using Pd(MeCN)_2_Cl_2_ as a catalyst (Scheme 23, reaction conditions 2). This coupling reaction was followed by the oxidation of the vinyl group of nucleoside **129** by OsO_4_ and acetylation of vicinal diol **130**. After deprotection of the 3',5'-hydroxyl groups, the 5'-hydroxyl group was dimethoxytritylated and the 3'-hydroxyl group phosphitylated to afford phosphoramidite **134**. The final phosphoramidite **134** was incorporated into oligodeoxynucleotide sequences via solid-phase synthesis by an automated DNA synthesizer.

**Scheme 24:**
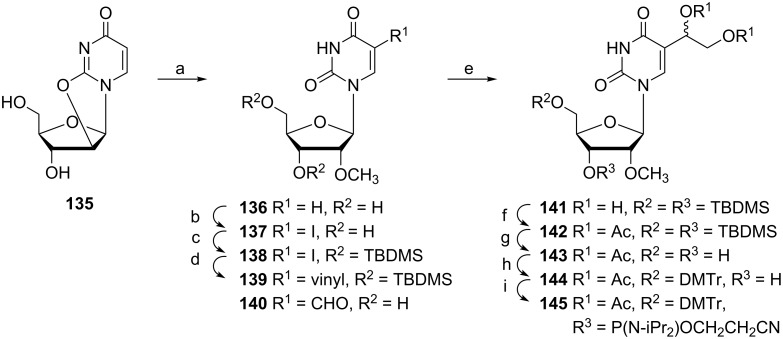
Synthesis of phosphoramidite **145**. (a) B(OCH_3_)_3_, CH(OCH_3_)_3_, Na_2_CO_3_, MeOH, 150 °C; (b) I_2_, (0.6 equiv), CAN (0.5 equiv), AcOH, 80 °C; (c) TBDMSCl (3.5 equiv), imidazole (5 equiv), DMF; (d) 5 mol % Pd(CH_3_CN)_2_Cl_2_, Bu_3_SnCH=CH_2_, CH_3_CN, 80 °C; (e) OsO_4_, NMO (2.5 equiv), acetone H_2_O *t*-BuOH (4:1:1); (f) Ac_2_O (4 equiv), pyridine; (g) TBAF (2 equiv), AcOH (2 equiv), THF; (h) DMTrCl (1.5 equiv), pyridine; (i) [(iPr)_2_N]_2_POCH_2_CH_2_CN (1.8 equiv), DCI (0.7 equiv), CH_3_CN CH_2_Cl_2_ (1:10).

Modified oligonucleotides can also serve as a tool for the investigation of interactions between NF-κB proteins (NF-κB is a protein complex that controls the transcription of DNA and plays a key role in regulating the immune response to infection). A study was reported by Kittaka and co-workers [38] which described an interaction between the above noted proteins and modified oligonucleotides, in which thymidine is replaced by a 5-formyl derivative. A phosphoramidite **145** for oligonucleotide synthesis was prepared from *O*^2^-2'-cyclouridine (**135**) by a multistep synthesis (Scheme 24). In a first step, *O*^2^-2'-cyclouridine (**135**) was selectively methylated at the 2'-*O* atom and subsequently iodinated at position 5 with CAN-I_2_ in AcOH to give nucleoside **137** in 74% yield. Protection of 3',5'-diol **137** by TBDMS groups (quantitative) afforded nucleoside **138** which was subsequently subjected to a Stille coupling reaction with tributyl(vinyl)tin using Pd(CH_3_CN)_2_Cl_2_ as a catalyst followed by oxidation with OsO_4_/NMO to afford the dihydroxy derivative **141** in 77% yield after two steps. The desired phosphoramidite **145** was obtained in 90% yield after acetylation of the vicinal diol **141**, selective deprotection of 3',5'-hydroxyl groups (**143** in 96% yield), dimethoxytritylation of the 5'-hydroxyl group (**144** in 89% yield) and finally, 3'-*O*-phosphitylation.

**Figure 8:**
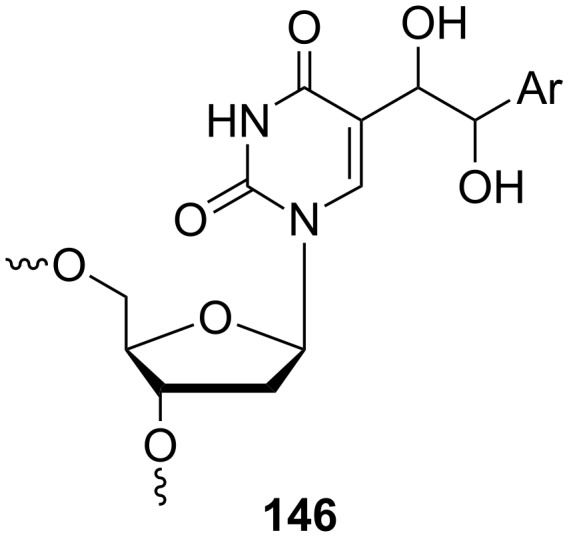
Oligonucleotide **146**.

An aryl moiety containing phosphoramidite, oligonucleotide **146** (Figure 8), was synthesized by Ding and co-workers and described its utilization of as a hole migration probe [39]. This compound should serve as a molecular probe that facilitates selective detection of excess electron transfer or hole migration in DNA using gel electrophoresis.

**Scheme 25:**
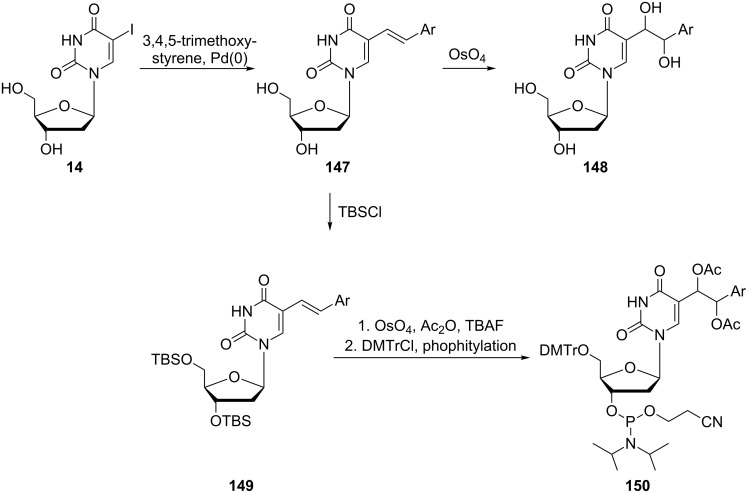
Synthesis of phosphoramidite **150**.

The synthesis of the desired phosphoramidite **150** (Scheme 25) started with the Pd-catalyzed cross-coupling of 5-iodo-2'-deoxyuridine (**14**) and a styrene to afford nucleoside **147**. The oxidation of alkene function in **147** with OsO_4_ led to a mixture of diastereomers of vicinal diols **148**. For the introduction of the oligodeoxynucleotide **146**, the dihydroxynucleoside **147** was converted to the corresponding phosphoramidite. This was carried out as follows. First, the hydroxyl groups of deoxyribofuranosyl moiety were silylated to give the protected nucleoside **149** which was then oxidized with OsO_4_ to afford the protected vicinal diol. The free hydroxyl groups attached to the side chain at position 5 of the uracil ring were acetylated and the silyl protection groups at the sugar ring were removed by the reaction with TBAF. Finally, the 5'-hydroxyl groups were tritylated and the 3'-hydroxy group converted to the corresponding phosphoramidite **150**. The resulting phosphoramidite was incorporated into a 12-mer oligodeoxynucleotide via automated solid-phase synthesis.

#### Synthesis of bis heterocyclic derivatives

**Figure 9:**
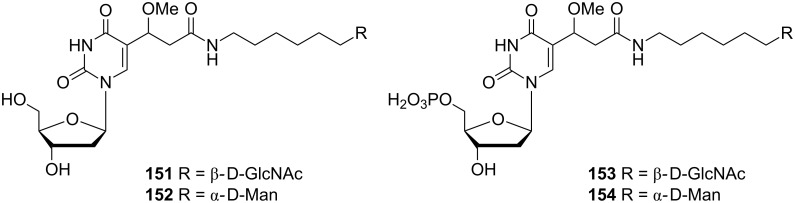
2'-Deoxyuridine derivatives **151**–**154**.

Sarfati and co-workers published an interesting and facile synthesis of C-5 alkylated 2'-deoxyuridine and uridine derivatives [40]. The C-5 position can be substituted by glycosides of either 2-acetamido-2-deoxy-β-D-glucopyranose or α-D-mannopyranose. All the products **151**–**154** (Figure 9) were formed as by**-**products of the palladium catalyzed addition reaction of alkenes to C-5-mercuriated deoxyuridines.

**Scheme 26:**
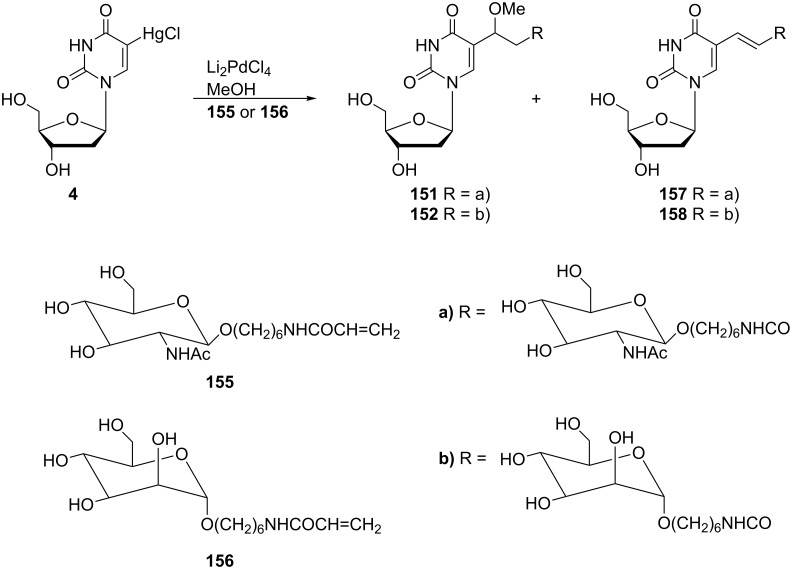
Synthesis of 2'-deoxyuridine derivatives **151**–**152**.

The synthesis of derivatives **151** and **152** started with condensation reactions of alkenes **155** and **156** with 5-chloromercuri-2'-deoxyuridine **4** in the presence of a palladium catalyst (Scheme 26).

The vinyl derivatives **157** and **158** were obtained as major products. However, methoxy derivatives **151** and **152** were produced in modest yields. The monophosphate derivatives **153** and **154** were formed by a similar reaction with mercuriated 2'-deoxyuridine monophosphates.

**Scheme 27:**
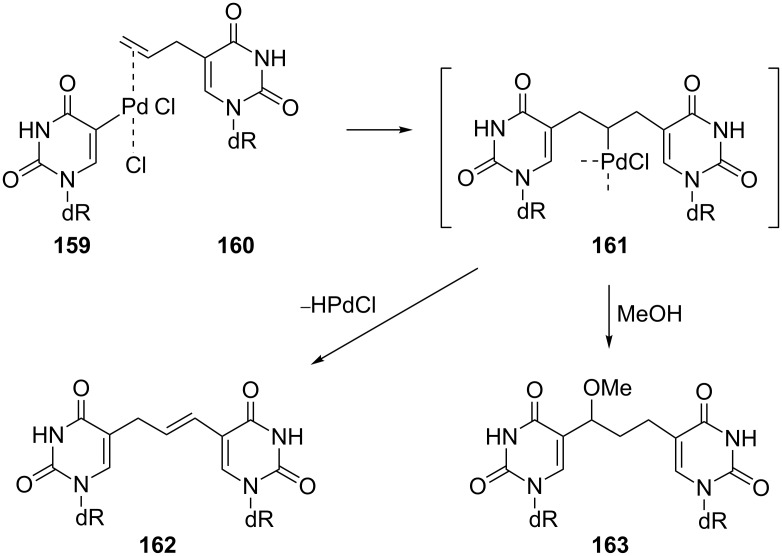
Synthesis of 5-[3-(2'-deoxyuridin-5-yl)-1-methoxyprop-1-yl]-2'-deoxyuridine (**163**).

Almost 10 years earlier, Bergstrom and co-workers published a synthesis based on the same reaction – the Heck cross-coupling reaction of an alkene with an organometallic derivative [41] in which two pyrimidine nucleosides were coupled (Scheme 27). Thus, 5-(chloromercuri)-2'-deoxyuridine was converted to its reactive palladium intermediate **159** by the reaction with 20 mol % of Li_2_PdCl_4_ in methanol. Consequently, allyl chloride reacted with this intermediate **159** and gave (*E*)-5-[3-(2'-deoxyuridin-5-yl)-1-propen-1-yl]-2'-deoxyuridine (**162**) as a major product with 5-[3-(2'-deoxyuridin-5-yl)-1-methoxyprop-1-yl]-2'-deoxyuridine (**163**) as a byproduct.

#### Synthesis of metallocenonucleosides

**Scheme 28:**
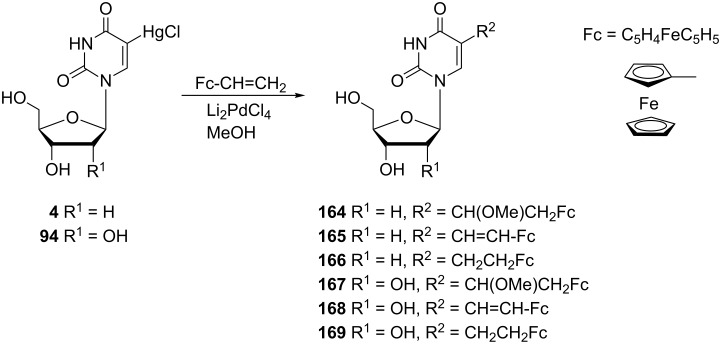
Synthesis of “metallocenonucleosides” **164** and **167**.

The first “metallocenonucleosides” were synthesized and characterized by Meunier and co-workers in 1991 [42]. The term “metallocenonucleosides” was derived from nucleosides containing a metallocene moiety and these compounds were prepared in order to study their chemical as well as cytotoxic properties (Scheme 28). The reported work was focused on a group of nucleosides with the formula: a) Ns–CH=CH–Fc or b) Ns–CH_2_–CH_2_–Fc, where Ns (= nucleoside) is either uridine (derivatives **168**, **169**) or 2'-deoxyuridine (derivatives **165**, **166**), and Fc is the abbreviation of ferrocene of molecular formula C_5_H_4_FeC_5_H_5_. From the reaction of 5-(chloromercuri)-nucleosides **4** or **94** with ethenylferrocene, methoxyderivatives **164** or **167** were also formed along with nucleosides **165**, **166**, **168** and **169**.

#### Synthesis of pseudouridines

**Scheme 29:**
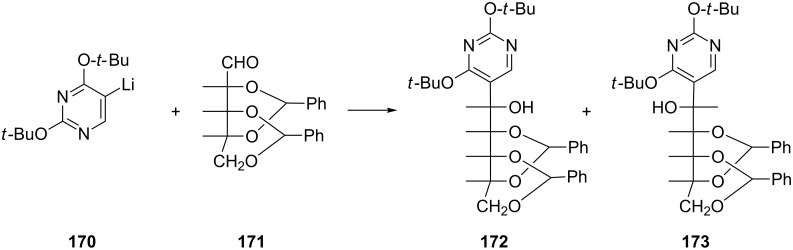
Synthesis of 5-(2,4:3,5-di-*O*-benzylidene-D-pentahydroxypentyl)-2,4-di-*tert*-butoxy-pyrimidine **172** and **173**.

Pseudouridine is a *C*-glycoside isomer of uridine and plays an important role in proteosynthesis. In organisms, pseudouridine is biosynthesized from uridine via the action of pseudouridine synthases. Nevertheless, the specific role of pseudouridines is still the subject of much research. In order to study pseudouridine analogues, many pseudouridine derivatives have been synthesized since 1961 [43–46]. Whilst all of these works achieved the synthesis of pseudouridines, the yields were not quite satisfactory. As late as 1971, Lerch and co-workers established reaction conditions and published advanced studies on the synthesis of pseudouridine (Scheme 29) [47]. Thus, 2,4-di-*tert*-butoxypyrimidin-5-yllithium (**170**) was reacted with 2,4:3,5-di-*O*-benzylidene-*aldehydo*-D-ribose (**171**) in tetrahydrofuran to afford a mixture of *allo* and *altro* isomers of 5-(2,4:3,5-di-*O*-benzylidene-D-pentahydroxypentyl)-2,4-di-*tert*-butoxy-pyrimidine **172** and **173**, respectively. A complete separation using preparative TLC afforded the *allo* isomer **172** in 25% yield and the *altro* isomer **173** in 37% yield.

**Figure 10:**
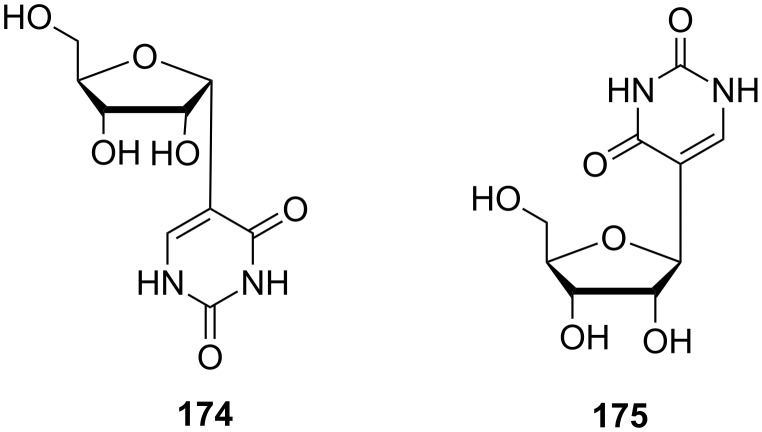
α- and β-pseudouridine (**174** and **175**).

**Figure 11:**
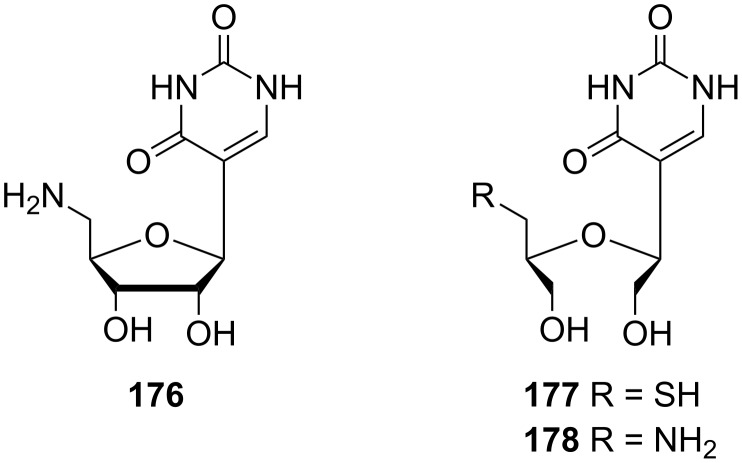
5'-Modified pseudouridine **176** and secopseudouridines **177**, **178**.

Subsequent cyclization of both isomers in hydrochloric acid gave α- and β-furanose forms of pseudouridine **174** and **175**, respectively (Figure 10). Other studies on the synthesis of pseudouridine analogues were made by Lee and co-workers 20 years later [48]. The 5'-modified pseudouridine **176** and secopseudouridines **177** and **178** were prepared via the ring cleavage of the sugar moiety (Figure 11).

### Biological activity

A number of the previously mentioned compounds were synthesized in order to evaluate their antiviral and cytotoxic activity. Moreover, antibacterial activity of some of these derivatives has also been studied. Some of the tested compounds have shown interesting results and a brief survey is given in the following section.

#### Antiviral activity

**Figure 12:**
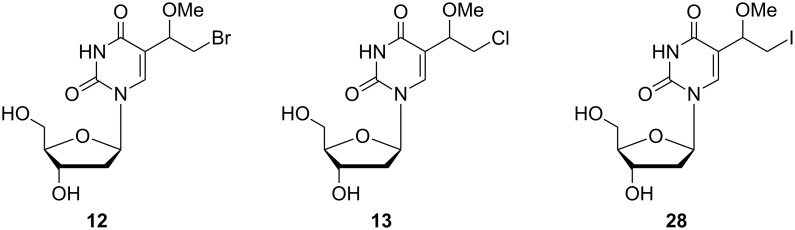
Methoxy derivatives **12**, **13** and **28**.

Shortly after the discovery of antiviral activity of 5-ethyl-2'-deoxyuridine [49], further C-5 modified analogues were synthesized and studied as potent antiviral agents. Some of the compounds prepared were 5-(1-methoxy-2-bromoethyl)-2'-deoxyuridine (**12**), 5-(1-methoxy-2-chloroethyl)-2'-deoxyuridine (**13**) [9,50] and 5-(1-methoxy-2-iodoethyl)-2'-deoxyuridine (**28**) [10] (Figure 12). These methoxy-haloethyluridines were tested against the *Herpes simplex* virus type 1 (HSV-1) and their activity compared with the antiviral activity of acyclovir and BVDU (5-(2-bromovinyl)-2'-deoxyuridine). The bromo derivative **12** exhibited greater activity than the corresponding chloro analogue **13**. Nevertheless, antiviral activity was weaker in comparison to acyclovir or BVDU. The most active iodo derivative **28** exhibited an antiviral activity approaching that of IVDU (5-(2-iodovinyl)-2'-deoxyuridine) and acyclovir.

**Figure 13:**
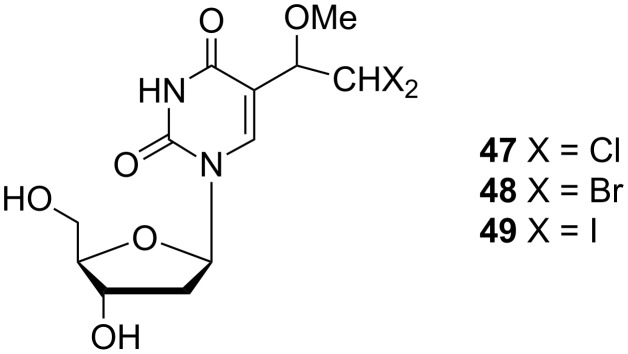
5-(1-Methoxy-2,2-dihaloethyl)-2'-deoxyuridines **47**–**49**.

The introduction of another halogen atom led to the preparation of 5-(1-methoxy-2,2-dihaloethyl)-2'-deoxyuridines **47**–**49** (Figure 13) [13]. All of these compounds were subjected to in vitro antiviral testing against HSV-1, HSV-2, VZV (*Varicella zoster* virus), HCMV (*Human cytomegalovirus*) and EBV (Epstein–Barr virus) and compared with the activity of 5-(1-hydroxydihaloethyl) analogues. In general, hydroxyl derivatives were more active than methoxy derivatives **47**–**49** against HSV-1, HSV-2, VZV and EBV. All of the investigated derivatives **47**–**49** were inactive against HCMV.

**Figure 14:**
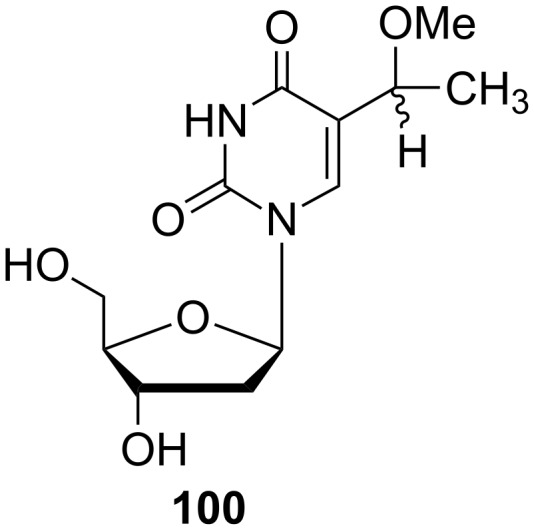
5-(1-Methoxyethyl)-2'-deoxyuridine **100**.

Another C-5 substituted 2'-deoxyuridine analogue, 5-(1-methoxyethyl)-2'-deoxyuridine (**100**), (Figure 14) was investigated as a potent antiviral agent against HSV-1, HSV-2 and HCMV [28]. The compound was as active as 5-ethyl-2'-deoxyuridine (EDU) against both HSV-1 and HSV-2 but less active against HCMV than EDU and ganciclovir.

**Figure 15:**
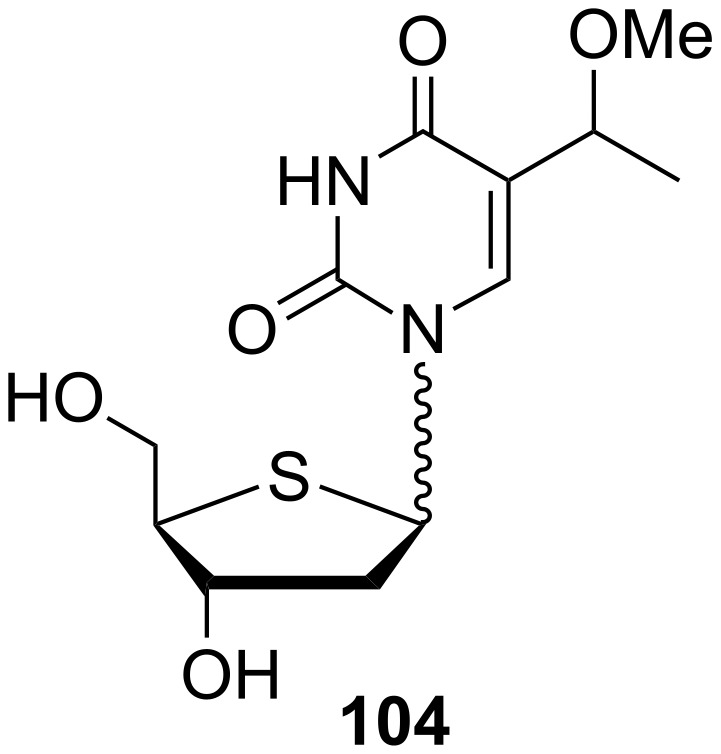
2'-Deoxy-5-(1-methoxyethyl)-4'-thiouridine (**104**).

The discovery of (*E*)-5-(2-bromovinyl)-2'-deoxy-4'-thiouridine (4'-S-BVDU) as a highly active agent against HSV-1, HSV-2 and VZV [29] inspired chemists to synthesize a group of 2'-deoxy-4'-thionucleosides [30]. In this context, anomeric 2'-deoxy-5-(1-methoxyethyl)-4'-thiouridine (**104**) (Figure 15) was prepared and its antiviral activity evaluated. However, this thio derivative **104** did not show any significant activity.

**Figure 16:**
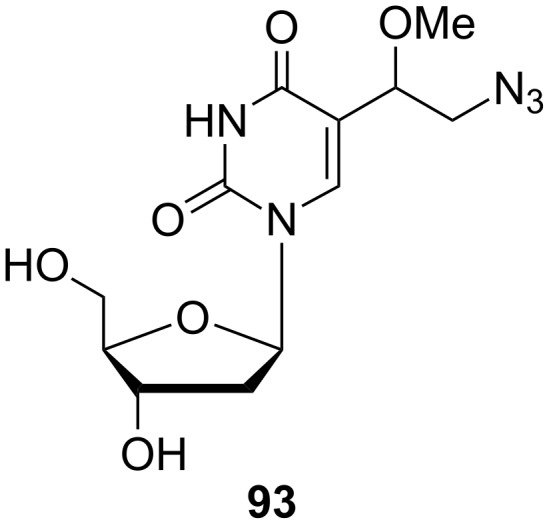
5-(1-Methoxy-2-azidoethyl)-2'-deoxyuridine (**93**).

Amongst the mentioned derivatives, azido nucleoside **93** (Figure 16) was prepared in order to determine antiviral activity against HSV-1, HSV-2, VZV and HCMV [24]. However, this compound also did not exhibit significant antiviral properties.

**Figure 17:**
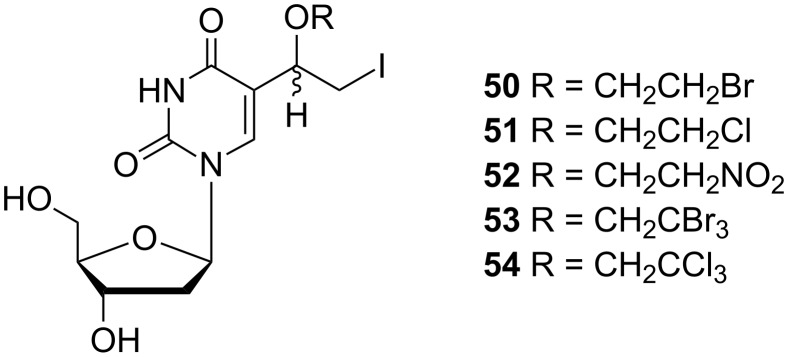
5-[1-(2-Halo(or nitro)ethoxy-2-iodoethyl)]-2'-deoxyuridines **50**–**54**.

Recent research dealing with new antiviral agents has been focused on the study of the antiviral activity of 5-[1-(2-halo(or nitro)ethoxy-2-iodoethyl)]-2'-deoxyuridines **50**–**54** (Figure 17) [16]. These nucleosides were evaluated in vitro for inhibitory activity against thymidine-kinase (TK) positive and negative strains of *Herpes simplex* virus type-1. All of these 2'-deoxyuridine analogues exhibited only weak anti-HSV-1 activity.

#### Cytotoxic activity

Only a few derivatives have been tested for their anticancer properties. The cytotoxic activity for derivatives **12**, **13** and **28** (Figure 12) were determined by an in vitro L1210 assay [9–10]. However, a comparison of the results for the investigated compounds with those of the reference compound melphalan showed lower activity.

**Figure 18:**
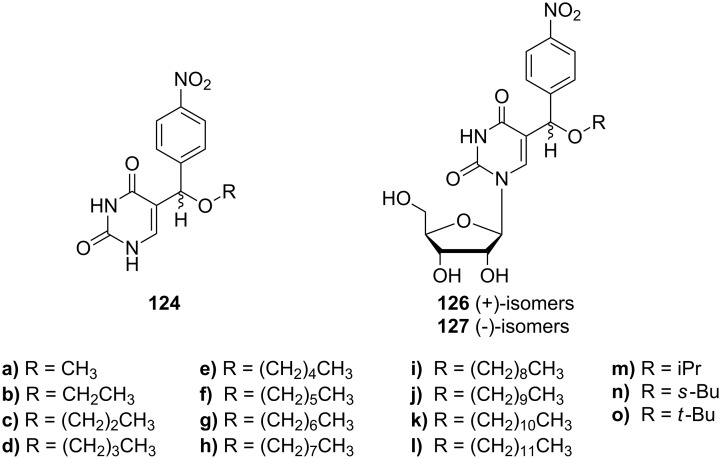
5-[Alkoxy-(4-nitrophenyl)-methyl] uracil analogues **124**, **126** and **127**.

Recent studies on cytotoxic activity of 5-[alkoxy-(4-nitrophenyl)methyl]uracil analogues **124, 126** and **127** (Figure 18) have been published [34–35]. All of the prepared compounds were tested for their cytotoxic activity in vitro against different cell lines and relationships between structure and cytotoxic activity were evaluated. Although all of the tested compounds exhibited weaker activity than reference carboplatin or 6-thioguanine, interesting relationships between activity and length of alkyl chain were observed.

#### Antibacterial activity

The recurrence of the chronic infectious disease tuberculosis has initiated research on new classes of antimycobacterial agents. The exigency of new drugs was also caused by multidrug-resistant tuberculosis strains, which are resistant to the most widely used agents, either Isoniazid or Rifampicin, and the need for new highly active compounds is increasing. Tuberculosis is caused by species of the genus *Mycobacterium,* for instance, *Mycobacterium tuberculosis*, *Mycobacterium avium* and *Mycobacterium bovis.*

**Figure 19:**
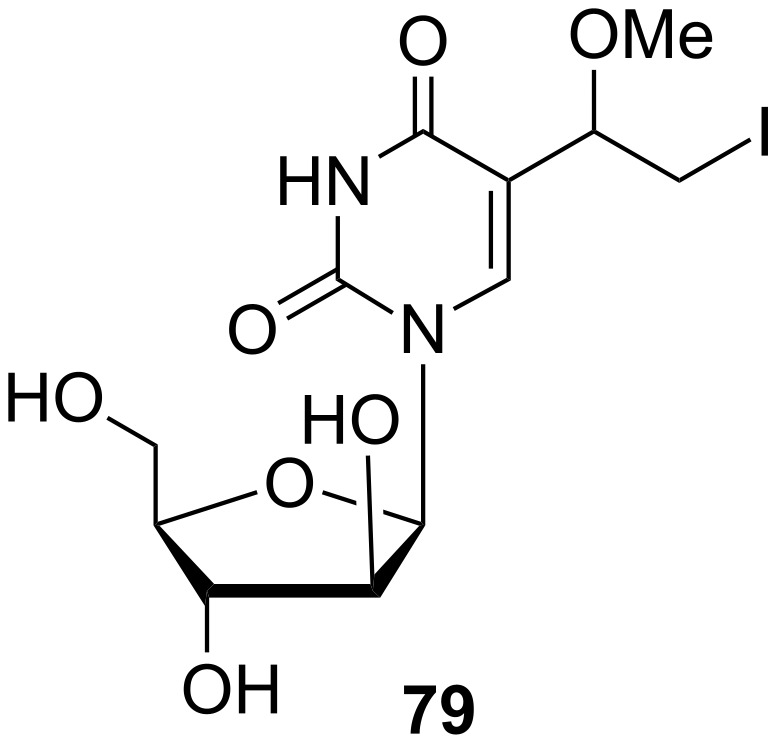
Methoxyiodoethyl pyrimidine nucleoside **79**.

Recently, a study on the effect of arabinofuranosyl analogues against *Mycobacterium* was published [19]. A series of 1-β-D-2'-arabinofuranosyl pyrimidine nucleosides was prepared in order to evaluate their antimycobacterial activity. Amongst others, the methoxyiodoethyl pyrimidine nucleoside **79** (Figure 19) was synthesized. Nevertheless, this nucleoside did not show any significant antimycobacterial activity.

In addition to this, nucleosides containing a dodecynyl moiety instead of an alkoxyhaloethyl group proved to be significantly active. The introduction of longer alkynyl chains might be a successful way to obtain potentially active antimycobacterial drugs.

**Figure 20:**
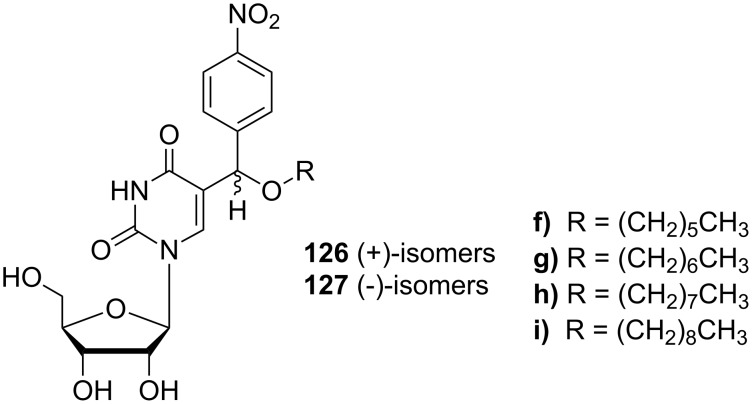
5-[alkoxy-(4-nitro-phenyl)-methyl]uridines **126** and **127**.

Recently, the antimicrobial activity of 5-[alkoxy-(4-nitrophenyl)methyl]uridines **126, 127** has been studied [35]. Eight isomers with different alkyl side chain lengths, **126** and **127** (Figure 20), were tested for their antimicrobial activity against standard reference gram-positive and gram-negative bacterial strains such as *Enterococcus faecalis* CCM 4224, *Staphylococcus aureus* CCM 3953*, Escherichia coli* CCM 3954 and *Pseudomonas aeruginosa* CCM 3955 and against gram-positive and gram-negative bacteria obtained from clinical material of patients treated at the University Hospital in Olomouc (methicillin resistant *Staphylococcus aureus* - MRSA, *Staphylococcus haemolyticus*, *Escherichia coli* and *Pseudomonas aeruginosa*) with resistance to currently used fluoroquinolones. Only the octyl and nonyl derivatives **126h**, **127h** and **126i**, **127i** showed slight activity against *Enterococcus faecalis* CCM 4224, *Staphylococcus aureus* CCM 3953, *Staphylococcus aureus* (MRSA) and *Staphylococcus haemolyticus*.

## Conclusion

This review was an attempt to summarize all available information on the synthesis and biological activity of selected C-5 substituted pyrimidine derivatives. Many authors have reported facile and successful syntheses by a large range of methods to obtain the desired compounds, and, in addition, they have also highlighted ineffectual synthetic routes. Most of the published derivatives were biologically inactive; although some exhibited weak activity. However, all of these results have made a significant and invaluable contribution to the development of new potent antiviral, cytotoxic or antibacterial agents and have elucidated possible structure–activity relationships.
